# Supplementary material for: How Many People Experience Unsafe Medical Care in Thailand, and How Much Does It Cost under Universal Coverage Scheme?
Source: Healthcare (Basel). 2023 Apr 13;11(8):1121. doi: 10.3390/healthcare11081121 (PMC10137567; doi:10.3390/healthcare11081121)
Supplement: Supplementary file 1 [file healthcare-11-01121-s001.zip › healthcare-2272077-supplementary.pdf]

Table S1. ICD-10 codes for patient safety indicators.

ICD-10 codes	Descriptions	Average number of medical harms between 2016-2020 (times)
A02	Salmonella enteritis	3,159
A04	Other bacterial intestinal infections	16,169
A41	Other sepsis	109,145
B95	Streptococcus and staphylococcus as the cause of diseases classified to other chapters	8,863
B96	Other specified bacterial agents as the cause of diseases classified to other chapters	55,260
B97	Viral agents as the cause of diseases classified to other chapters	2,231
E15	Nondiabetic hypoglycaemic coma<br>(Incl. Drug-induced insulin coma in nondiabetic, Hyperinsulinism with hypoglycaemic coma, Hypoglycaemic coma NOS)	5,533
E16.0	Drug-induced hypoglycaemia without coma	8,507
E27.3	Drug-induced adrenocortical insufficiency	318
E89	Postprocedural endocrine and metabolic disorders, not elsewhere classified	2,241
G97	Postprocedural disorders of nervous system, not elsewhere classified	316
H59	Postprocedural disorders of eye and adnexa, not elsewhere classified	1,998
I26	Pulmonary embolism	5,565
I82	Other venous embolism and thrombosis	2,100
J95	Postprocedural respiratory disorders, not elsewhere classified	15,762
K91	Postprocedural disorders of digestive system, not elsewhere classified	2,335
L89	Decubitus ulcer and pressure area	40,839
M96	Postprocedural musculoskeletal disorders, not elsewhere classified	1,217
N99	Postprocedural disorders of genitourinary system, not elsewhere classified	710
O29	Complications of anaesthesia during pregnancy	15
O70	Perineal laceration during delivery	16,123
O71	Other obstetric trauma	1,823
O74	Complications of anaesthesia during labour and delivery	39
O75	Other complications of labour and delivery, not elsewhere classified	3,948
O85	Puerperal sepsis	663
O86	Other puerperal infections	1,623
O89	Complications of anaesthesia during the puerperium	34
O90	Complications of the puerperium, not elsewhere classified	851
P03	Fetus and newborn affected by other complications of labour and delivery	12,197
P12	Birth injury to scalp	6,921
P13	Birth injury to skeleton	460
P14	Birth injury to peripheral nervous system	350
P15	Other birth injuries	560
T36	Poisoning by systemic antibiotics	2,931
T37	Poisoning by other systemic anti-infectives and antiparasitics	585
T38	Poisoning by hormones and their synthetic substitutes and antagonists, not elsewhere classified	470
T39	Poisoning by nonopioid analgesics, antipyretics and antirheumatics	5,167
T41	Poisoning by anaesthetics and therapeutic gases	48
T42	Poisoning by antiepileptic, sedative-hypnotic and antiparkinsonism drugs	3,111
T42.7	Antiepileptic and sedative-hypnotic drugs, unspecified	348
T43	Poisoning by psychotropic drugs, not elsewhere classified	2,521
T44	Poisoning by drugs primarily affecting the autonomic nervous system	587
T45	Poisoning by primarily systemic and haematological agents, not elsewhere classified	5,580
T45.5	Anticoagulants	3,821
T45.7	Anticoagulant antagonists, vitamin K and other coagulants	17

ICD-10 codes	Descriptions	Average number of medical harms between 2016-2020 (times)
T46	Poisoning by agents primarily affecting the cardiovascular system	632
T47	Poisoning by agents primarily affecting the gastrointestinal system	193
T48	Poisoning by agents primarily acting on smooth and skeletal muscles and the respiratory system	272
T49	Poisoning by topical agents primarily affecting skin and mucous membrane and by ophthalmological, otorhinolaryngological and dental drugs	565
T50	Poisoning by diuretics and other and unspecified drugs, medicaments and biological substances	2,801
T80	Complications following infusion, transfusion and therapeutic injection	4,350
T81	Complications of procedures, not elsewhere classified (ex. Haemorrhage and haematoma complicating a procedure, Shock during or resulting from a procedure, Disruption of operation wound, Infection following a procedure, Foreign body accidentally left in body cavity or operation wound following a procedure etc.)	34,356
T82	Complications of cardiac and vascular prosthetic devices, implants and grafts	5,969
T83	Complications of genitourinary prosthetic devices, implants and grafts	2,036
T84	Complications of internal orthopaedic prosthetic devices, implants and grafts	6,385
T85	Complications of other internal prosthetic devices, implants and grafts	16,015
T87	Complications peculiar to reattachment and amputation	1,014
T88	Other complications of surgical and medical care, not elsewhere classified	6,762

Table S2. Inpatient category of Thailand's consumer price index (CPI).

Year	CPI
2016	99.69
2017	99.92
2018	99.99
2019 (base year)	100.00
2020	100.05

Table S3. Characteristics of inpatient visit.

	2016		2017		2018		2019		2020	
	Number	%	Number	%	Number	%	Number	%	Number	%
**Total visits**	6,056,500	100	6,006,660	100	6,264,661	100	6,366,463	100	5,758,165	100
**Age (years)**										
mean ± SD	39.14 ± 28.33		40.17 ± 28.21		40.11 ± 28.35		41.11 ± 28.23		43.09 ± 27.67	
**Age group**										
0-14	1,677,053	27.69	1,575,436	26.23	1,691,317	27.00	1,646,175	25.86	1,283,621	22.29
15-59	2,483,391	41.00	2,483,816	41.35	2,530,714	40.40	2,562,273	40.25	2,404,989	41.77
≥ 60	1,896,056	31.31	1,947,408	32.42	2,042,630	32.61	2,158,015	33.90	2,069,555	35.94
**Sex**										
Female	2,881,668	47.58	2,873,017	47.83	3,024,824	48.28	3,088,615	48.51	2,790,766	48.47
Male	3,174,832	52.42	3,133,643	52.17	3,239,837	51.72	3,277,848	51.49	2,967,399	51.53
**Hospital types**										
Center hospital	1,328,500	21.94	1,332,033	22.18	1,366,387	21.81	1,380,172	21.68	1,281,493	22.26
General hospital	1,392,396	22.99	1,406,682	23.42	1,464,010	23.37	1,479,134	23.23	1,363,341	23.68
Community hospital	2,627,680	43.39	2,597,254	43.24	2,767,683	44.18	2,843,502	44.66	2,511,310	43.61
Private hospital	201,867	3.33	175,249	2.92	165,714	2.65	162,042	2.55	132,336	2.30
Others (ex. medical university hospitals, health center)	506,057	8.36	495,442	8.25	500,867	8.00	501,613	7.88	469,685	8.16
